# Overview of recent advances in zearalenone (ZEN) research

**DOI:** 10.17179/excli2026-9422

**Published:** 2026-07-17

**Authors:** Gabriela Pilarska, Magdalena Twaruzek

**Affiliations:** 1 Department of Physiology and Toxicology, Faculty of Biological Sciences, Kazimierz Wielki University, Chodkiewicza 30, 85-064 Bydgoszcz, Poland

**Keywords:** mycotoxin, toxicology, modified mycotoxins, food safety, risk assessment, endocrine disruption

## Abstract

Zearalenone (ZEN) is a *Fusarium* derived mycotoxin widely detected in cereals and cereal-based products and recognized for its pronounced estrogenic activity. Although the fundamental aspects of ZEN chemistry, metabolism, and toxicological effects have been well described, recent research has revealed a more complex picture of its biological significance and exposure pathways. Increasing attention has been devoted to biomonitoring approaches that allow the assessment of internal exposure rather than relying solely on contamination levels in food or feed matrices. At the same time, studies on modified and masked forms of ZEN suggest that these derivatives may represent an additional and often underestimated source of exposure. Recent findings also indicate that the toxicological impact of ZEN extends beyond endocrine disruption and may involve effects on intestinal barrier integrity, metabolic regulation, and inflammatory processes. Furthermore, advances in analytical techniques and research on enzymatic degradation and inhibition of *Fusarium* biosynthesis are expanding possibilities for more effective monitoring and mitigation strategies. This review summarizes recent developments in the understanding of ZEN occurrence, metabolism, toxicity, and detection, highlighting emerging research directions relevant to food safety, animal health, and modern exposure assessment.

See also the graphical abstract(Fig. 1).

## 1. Introduction

Zearalenone (ZEN) remains one of the most important mycotoxins produced by *Fusarium* fungi and, at the same time, one of the best-recognised substances with oestrogen-like effects present in food and feed. The 2021 article by Ropejko and Twarużek accurately summarised the basic information on the chemical characteristics of ZEN, its main metabolites, its occurrence in plant products and the basic mechanisms of toxicity (Ropejko and Twarużek, 2021[36]). In subsequent years, the picture of the problem has not changed significantly, but it has become much more complex. Firstly, the importance of biomonitoring, which allows not only the contamination of the matrix to be assessed, but also the actual exposure of the organism, has clearly increased. Secondly, a growing number of studies concern modified forms, including glucosides and other derivatives, which may constitute a reservoir of toxicity. Thirdly, the importance of ZEN for the integrity of the intestinal barrier, crypt stem cell function, metabolism and inflammatory response, and not only for the reproductive system, is being highlighted more strongly than before. Fourthly, research is developing on limiting ZEN biosynthesis by *Fusarium* and on enzymatic detoxification of the toxin itself (EFSA, 2016[8]; Falkauskas et al., 2022[12]; Fang et al., 2022[14]; Ruan et al., 2022[37]; Stakheev et al., 2022[38]; Wu et al., 2022[43]; Fruhauf et al., 2024[15]; Gomes et al., 2025[18]; Huo et al., 2025[23]; Lisieska-Żołnierczyk et al., 2025[28]; Xu et al., 2025[44]).

In light of current data, ZEN should be treated as a multi-layered problem, including contamination of cereals and feed, metabolic changes in animals and humans, co-occurrence of other mycotoxins, the impact of climatic conditions on the production of toxin producing fungi, and the quality of analytical tools used to assess exposure. It is becoming increasingly clear that testing a single batch of grain or a single feed sample does not reflect the full biological risk unless it is combined with biomonitoring data, an assessment of modified metabolites, and knowledge of the sensitivity of a given species or population group. The aim of this study is to expand on the findings of the 2021 review by discussing the most important research directions in recent years and highlighting their significance for food and feed safety, animal health and laboratory practice.

In classic studies, zearalenone is primarily presented as non-steroidal substance with oestrogen-like properties, synthesised by selected *Fusarium* species and present mainly in cereals and cereal products. This approach remains true, but is now insufficient. This statement complements the multi-layered framework described above by emphasizing its biological and environmental integration (EFSA, 2016[8]; Ropejko and Twarużek, 2021[36]; Huo et al., 2025[23]). The 2021 article by Ropejko and Twarużek remains valuable as a starting point for further consideration, as it provides a concise summary of information on the origin of ZEN, its biotransformation to α-zearalenol and β-zearalenol, the basis for its occurrence in food, and the key importance of oestrogenicity for toxic effects (Ropejko and Twarużek, 2021[36]). However, from the perspective of the current state of knowledge, it is clear that four areas have developed particularly since then. The first concerns the transition from monitoring matrix contamination to monitoring actual internal exposure. The second covers the increasing importance of modified forms and secondary metabolites. The third relates to a deeper understanding of toxicity, especially in the context of the intestine, metabolism and inflammatory response. The fourth relates to attempts to influence the biosynthesis of ZEN itself and the intensive development of technologies for its degradation (Falkauskas et al., 2022[12]; Fang et al., 2022[14]; Ruan et al., 2022[37]; Stakheev et al., 2022[38]; Fruhauf et al., 2024[15]).

As a result, today's view of ZEN is more systematic than it was a few years ago. In practice, it is no longer just a matter of answering the question of whether a given batch of grain contains zearalenone above the regulatory threshold. Increasingly, the question of the extent to which a given exposure translates into actual exposure, what metabolites appear in the body, how long the biological effects persist, and whether the toxin acts alone or rather as part of a mixture of mycotoxins is becoming more important. This shift in perspective is of fundamental importance for modern food toxicology.

## 2. What Remains Relevant from the 2021 Review

Although this study is an update, it is worth first pointing out which conclusions from the 2021 review remain fully relevant. Firstly, it remains undisputed that ZEN is one of the most important *Fusarium* mycotoxins present in maize, wheat, barley, oats and derived products (Ropejko and Twarużek, 2021[36]). Secondly, its main biological effect remains oestrogen-like activity, although it is now more strongly emphasised than before that this does not exhaust the entire spectrum of its toxic effects. Thirdly, the distinction between the parent compound and its metabolites, especially α-zearalenol, which in many models exhibits a stronger oestrogenic effect than ZEN itself, remains valid (EFSA, 2011[9], 2017[10]; Ropejko and Twarużek, 2021[36]).

The emphasis placed in the original article on the importance of the entire food and feed chain remains equally relevant. Zearalenone contamination is not a random or purely laboratory phenomenon. It results from a combination of factors including weather conditions, plant variety, fungal infection, harvesting methods, transport, storage and processing. This is why ZEN is one of those mycotoxins whose presence is not only a parameter of product quality, but also an indirect indicator of specific disruptions in the production and storage system of plant raw materials (Ropejko and Twarużek, 2021[36], The European Commission, 2023[40]).

After 2021, however, what has changed most is how broadly contamination data is interpreted. Increasingly, concentrations in food or feed are treated as initial information rather than final information. In practice, this means that product measurement should be supplemented by an assessment of masked forms, an analysis of co-occurring mycotoxins, and, in health-oriented studies, biomonitoring.

## 3. Current Legal Framework and Importance of Risk Assessment

In the regulatory sphere, EFSA opinions on zearalenone in food, its modified forms and risks to animal health remain an important point of reference. In assessing the risk to humans, the tolerable daily intake established by EFSA and subsequently developed into a group approach that also covers modified forms remains crucial (EFSA, 2011[9], 2016[8]; The European Commission, 2023[40]). In practice, this means that it is increasingly difficult to justify treating certain ZEN derivatives as completely separate and biologically insignificant, since some of them may be converted into active forms.

From a legal perspective, Commission Regulation (EU) 2023/915 has become an important reference point for food safety, as it has streamlined the system of maximum levels for certain contaminants in food, including zearalenone (The European Commission, 2023[40]). The significance of this act is not limited to legislative change. For laboratory and control practices, it means the consolidation of the conformity assessment framework and a clearer anchoring of requirements for specific product categories. However, it should be emphasised that the compliance of a batch with the applicable limit does not always exhaust the issue of biological safety. This is particularly true in situations where exposure is chronic, involves vulnerable population groups or occurs in the presence of other fungal toxins.

In its risk assessment for animals, EFSA drew attention to significant species differences. Pigs in particular remain a species that is especially sensitive to the oestrogenic effects of ZEN, while other species may respond differently in terms of critical dose, metabolite profile and clinical picture (EFSA, 2017[10]). This observation continues to be of practical importance, as it reminds us that the same ZEN content in feed does not necessarily correspond to the same level of biological risk for all animals. Modern risk management therefore requires moving away from a completely universal interpretation model.

Recent monitoring studies conducted between 2021 and 2025 confirm that ZEN contamination remains widespread in both food and feed commodities. Large-scale surveys of feed and raw materials have reported detection frequencies ranging from approximately 20 % to over 50 %, depending on the commodity and geographic region (Gruber-Dorninger et al., 2025[20]). Updated monitoring reports published in 2025 further indicate that the global prevalence of ZEN in feed materials continues to remain high, particularly in maize-based commodities (EEA, 2025[7]).

In this context, preventing fungal infection during crop production remains one of the most effective strategies for reducing ZEN contamination. Agricultural practices such as crop rotation, the use of resistant crop varieties and proper storage conditions play a crucial role in minimizing fungal growth. Recent research has highlighted the importance of integrated crop management strategies aimed at limiting *Fusarium* infection and subsequent mycotoxin production. These approaches include optimized irrigation regimes, appropriate fungicide use and improved post-harvest storage conditions (Casu et al., 2024[2]). Climate change may additionally influence the effectiveness of these preventive measures, as changes in temperature and humidity patterns can alter fungal growth dynamics and mycotoxin production.

## 4. The Presence of ZEN in Food and Feed after 2021

After 2021, there has been no revolutionary change in the overall picture of ZEN occurrence, but more recent studies better show that actual exposure is often widespread, albeit generally at low doses. This empirical evidence supports the systematic view mentioned earlier, indicating that exposure is not limited to occasional exceedances but instead reflects the chronic presence of moderate concentrations across the food chain. The literature increasingly emphasises that it is precisely this chronic subclinical exposure that may be biologically significant, especially in combination with other stressors or in individuals and animals more susceptible to endocrine and metabolic disorders (EFSA, 2017[10]; Ropejko and Twarużek, 2021[36]).

Maize remains one of the cereal crops most susceptible to contamination by *Fusarium* species and therefore represents a major source of zearalenone (ZEN) in the global food and feed chain. Infection by toxigenic *Fusarium* fungi typically occurs during crop growth and may continue during post-harvest storage, leading to the accumulation of several mycotoxins, including ZEN, deoxynivalenol and fumonisins. These toxins frequently co-occur in maize commodities, increasing the potential risks to food and feed safety (Zheng et al., 2024[45]).

Recent studies confirm that ZEN contamination of maize remains widespread across different geographic regions, although its prevalence and concentration vary considerably depending on climatic conditions and agronomic practices. Environmental factors such as temperature, precipitation and humidity during key growth stages strongly influence *Fusarium* infection and mycotoxin production. While these causal claims are well-established, recent data shows that increased rainfall and humidity during flowering and grain development specifically correlate with higher contamination levels in maize (Chhaya et al., 2024[4]). This strengthens the argument for more flexible monitoring based on risk analysis rather than solely on a fixed, routine sampling plan.

For example, studies on cereal products available on the Polish market have shown that exceedances of permissible levels may not be frequent, but they do occur and cannot be treated as a marginal phenomenon. Moreover, even where the average consumer exposure does not exceed levels considered unsafe, there remains the question of individual variability, cumulative consumption of different products and the presence of masked forms that are not always routinely labelled. From a public health perspective, therefore, a 'constant background exposure' model is more realistic than a model based solely on the detection of rare batches of heavily contaminated products.

Furthermore, recent monitoring and review studies indicate that climate change may further influence the geographical distribution and prevalence of *Fusarium* mycotoxins in cereal production systems. Rising temperatures, increased precipitation, rainfall variability and episodes of high moisture during ripening and harvesting can create environmental conditions favourable for fungal growth and toxin biosynthesis, potentially increasing the risk of ZEN contamination in maize based food and feed commodities. This does not automatically mean a uniform increase in concentrations in all regions, but it strengthens the argument for more flexible monitoring based on risk analysis rather than solely on a fixed, routine sampling plan. For the agri-food industry, this means that mycotoxin monitoring needs to be more closely linked to agrometeorological data and the history of the raw material (Hudu et al., 2024[22]).

## 5. From Matrix Contamination to Internal Exposure

One of the most significant advances in recent years has been the development of biomonitoring. From a methodological point of view, its advantage over classic food or feed testing is that it reflects not only the presence of toxins in the external environment, but also their actual fate in the body: absorption, distribution, metabolic transformations and excretion. Thanks to biomonitoring, it is possible to move from the question 'is a given sample contaminated?' to the question 'how strong was the actual exposure of a given organism and what metabolites were produced?' (Falkauskas et al., 2022[12]; Chen et al., 2023[3]).

The study by Falkauskas et al. provided strong evidence for the usefulness of serum, urine and milk in assessing the exposure of dairy cows to zearalenone and its metabolites (Falkauskas et al., 2022[12]). The very fact that these compounds can be detected in several biological matrices is significant. It shows that the picture of exposure is not one-dimensional. One matrix may better reflect recent exposure, another chronic exposure, and yet another may be useful from the point of view of food safety of animal origin. In breeding practice, biomonitoring can therefore play a dual role: diagnostic and control.

This aspect is particularly relevant in the context of the transfer of zearalenone along the food chain. Although ZEN contamination occurs mainly in plant-derived products, it can also be detected in foods of animal origin due to carry-over from contaminated feed. ZEN and its metabolites may be transferred to milk, eggs and animal tissues; however, transfer rates are generally low because the toxin is extensively metabolized and rapidly excreted in animals (Osaili et al., 2022[33]). Recent studies have confirmed that ZEN and its metabolites can be detected in dairy milk, although concentrations are typically low and rarely exceed regulatory thresholds. Monitoring studies indicate that the transfer of mycotoxins from feed to milk is generally limited and depends on factors such as exposure level, animal metabolism and feeding practices (Ghanati et al., 2024[17]; González-Jartín et al., 2024[19]). These findings suggest that although animal-derived foods are not the primary source of human exposure to ZEN, contaminated feed may still contribute to indirect dietary exposure through the food chain.

A study by Wu et al., which assessed the impact of natural exposure of dairy cows to mycotoxins present in mouldy diet components, is of similar value (Wu et al., 2022[43]). The authors showed that exposure was associated not only with the presence of specific compounds, but also with changes in biochemical parameters and small molecule metabolism pathways in milk. This is an important conclusion because it emphasises that fungal toxins are not just 'contaminants' but biological factors capable of modifying the functioning of the entire organism. This is particularly important in animal production, where even moderate but chronic disorders can affect productivity, health and raw material quality.

In humans, the development of LC-MS/MS methods for multi-mycotoxin determination in urine has paved the way for a more accurate assessment of environmental exposure to mycotoxins, including ZEN (Chen et al., 2023[3]). The significance of such methods goes beyond purely laboratory innovation. They allow for the creation of more realistic population exposure models, the study of individual variation, and the analysis of the relationship between exposure and clinical and metabolic characteristics.

While LC-MS/MS remains the reference technique for biomonitoring studies, alternative rapid screening technologies are also being developed for the detection of mycotoxins in food and environmental samples.

In recent years, biosensor-based detection systems have emerged as promising tools for the rapid detection of mycotoxins, including zearalenone (ZEN). These devices combine biological recognition elements with physicochemical transducers, enabling sensitive and selective identification of target analytes in food matrices (Tang et al., 2023[39]).

Among the developed platforms, electrochemical and aptamer-based biosensors have attracted particular attention due to their high sensitivity, relatively low cost and rapid response. These sensors typically use antibodies or aptamers immobilized on electrode surfaces to generate measurable electrochemical signals in the presence of ZEN (Tang et al., 2023[39]).

Recent advances also highlight the role of nanomaterials such as gold nanoparticles, graphene and quantum dots in improving signal amplification and lowering detection limits, making biosensor-based systems promising tools for rapid food safety screening (Li et al., 2024[27]).

## 6. ZEN, Zeranol and Interpretative Difficulties in Bovine Urine

A particularly interesting field of research has been the distinction between natural feed exposure and the illegal use of zeranol. The study by Gomes et al. showed that the analysis of resorcylic acid lactones in bovine urine can be used to distinguish between the two situations, provided that the quantitative and interpretative approach is appropriate (Gomes et al., 2025[18]). This issue is important not only for toxicology, but also for veterinary legal supervision and production integrity control.

The essence of the problem is that some compounds appearing in animal urine may result both from natural transformations of resorcylic lactones originating from contaminated feed and from the administration of zeranol as a growth promoter. The mere presence of the metabolite is therefore not sufficient evidence of misuse. This study shows that biomonitoring requires not only sensitive analytical methods, but also mature interpretation algorithms. Otherwise, there is a risk of misattributing the origin of the detected compounds.

In a broader perspective, this is also important for the assessment of ZEN exposure itself. It shows that the metabolite profile in the body is the result of a dynamic interplay of metabolic processes, rather than a simple reflection of feed composition. This reinforces the argument that the modern interpretation of biomonitoring results should be cautious, contextual and based on knowledge of the physiology of the species.

## 7. Modified and Masked Forms: A Bigger Problem than Previously Thought

One of the most important interpretative shifts in recent years is the increase in the importance of modified forms of zearalenone. In the past, they were sometimes treated as derivatives of uncertain biological significance. Currently, more and more data indicate that some of them may act as a specific reservoir of toxicity, releasing the active parent compound under appropriate biological conditions (EFSA, 2016[8]; Ruan et al., 2022[37]).

Recent research has highlighted that such modified or “masked” forms are commonly produced during plant metabolism through conjugation reactions such as glucosylation or sulfation. These derivatives, including zearalenone-14-glucoside and sulfate conjugates, frequently co-occur with the parent toxin in contaminated cereals and may represent a substantial proportion of total ZEN related compounds detected in food matrices (Daud et al., 2023[6]; Nešić et al., 2023[32]).

A good example is zearalenone-14-glucoside. A study by Ruan et al. showed that in KGN cells, it can be hydrolysed to ZEN under the influence of β-glucosidase present in the extracellular matrix, and then cause intracellular toxicity (Ruan et al., 2022[37]). In practice, this means that a form that initially appears less active may regain its toxic potential in the body. This mechanism is important for safety assessment because it indicates the possibility of underestimating actual exposure if only free ZEN is measured.

In a broader sense, the problem of modified forms undermines the simplified model in which every chemical transformation is automatically equated with detoxification. In reality, the significance of a given derivative depends on whether it is stable, whether it can be hydrolysed, whether it binds to relevant biological structures, and what the tissue context of its action is. This is why EFSA's group approach to the health reference value for ZEN and its modified forms should be considered methodologically sound (EFSA, 2016[8]).

## 8. Toxicity beyond the Oestrogenic Effect

Although oestrogen like activity remains a central aspect of ZEN toxicity, a growing body of research indicates that it would be a mistake to reduce its effects solely to the reproductive axis. Contemporary literature increasingly describes ZEN as a compound that also affects oxidative stress, mitochondrial function, inflammatory response, cell membrane integrity, protein homeostasis and cell survival. In practice, this means that the oestrogenic effect is only one layer of biological action, although from a regulatory point of view it remains the most tangible and best documented (EFSA, 2011[9]; Fu et al., 2022[16]; Wang et al., 2024[42]; Lv et al., 2025[29]).

Several studies additionally indicate that these effects are closely associated with mitochondrial dysfunction and the activation of intracellular signaling pathways such as MAPK, PI3K/Akt and NF-κB, which regulate inflammation, apoptosis and cellular stress responses (Rai et al., 2020[35]; Lv et al., 2025[29]).

Of particular note are experimental studies showing that ZEN exacerbates oxidative and apoptotic damage in various cell types. For example, it has been shown that rutin can alleviate ZEN-induced damage in piglet endometrial stromal cells, which indirectly confirms the involvement of oxidative stress and the p53 pathway in the toxic mechanism (Wang et al., 2024[42]). In turn, in bovine mammary gland epithelial cells, ZEN can exacerbate LPS-induced oxidative stress and endoplasmic reticulum stress, accelerating apoptosis (Fu et al., 2022[16]). These results are important because they show the possibility of ZEN interacting with other biological factors, rather than acting in isolation. These processes are often accompanied by increased production of reactive oxygen species and disruption of mitochondrial membrane potential, which further amplifies apoptotic signaling cascades (Li et al., 2025[26]).

This means that the assessment of the risk associated with ZEN should take into account the tissue and disease context. The same level of exposure may have different meanings in a healthy organism and in an organism affected by inflammation, infection, metabolic stress or other mycotoxins. This approach better reflects real life conditions than the classic model of a single toxin tested in isolation.

## 9. The Intestine and Crypt Stem Cells as a New Area of Interest

The effect of mycotoxins on the intestinal epithelium has become a particularly important area of research in recent years. The article by Huo et al. presents a broad synthesis of knowledge on the impact of mycotoxins on the intestinal barrier, with particular emphasis not only on damage to tight junctions and increased permeability, but also on the dysfunction of crypt stem cells responsible for epithelial regeneration (Huo et al., 2025[23]).

This shift in perspective is also important for interpreting the action of ZEN.

In the past, the intestine was mainly treated as a site of contact with toxins and their absorption. Nowadays, it is increasingly seen as a target organ in which chronic exposure can disrupt not only the integrity of the barrier but also its regenerative potential. If a mycotoxin impairs the function of stem cells, the effects of exposure may persist longer than the contact with the toxin itself. The damage then becomes not only direct but also secondary, as the body is less able to rebuild the damaged epithelium (Huo et al., 2025[23]).

This is also important because the intestine does not function in isolation. Loss of intestinal barrier integrity can promote the translocation of bacterial components, activation of the immune system, changes in the microbiota and further exacerbation of inflammation. In this context, ZEN ceases to be solely a hormonal factor and becomes an element capable of sustaining broader dysregulation of the gut-immune metabolism axis. This interpretation seems particularly relevant in studies of chronic, low-dose exposure.

## 10. ZEN and the Metabolic Profile of Patients with Colorectal Cancer

An interesting, albeit cautiously interpreted, line of research is represented by a study on the impact of environmental exposure to ZEN on the metabolic profile of patients with sigmoid colon cancer and colorectal cancer on the day of hospital admission (Lisieska-Żołnierczyk et al., 2025[28]). The authors showed that the presence of ZEN was associated with specific differences in biochemical and metabolic parameters, including liver function, protein and lipid metabolism indicators.

Of course, studies of this kind do not allow us to claim that ZEN is the cause of colorectal cancer. Their significance is different. They show that environmental exposure to mycoestrogen may co-occur with measurable metabolic changes in patients with severe chronic disease. This opens the door to further research into whether fungal toxins can modify disease progression, metabolic profile, inflammatory response or treatment tolerance. Clinically, this is still a preliminary direction, but scientifically very interesting, as it combines environmental toxicology with oncology and metabolomics.

In a broader perspective, this type of research reminds us that ZEN does not have to be the primary causative factor to be biologically significant. It can act as a modifier of the body's condition, exacerbating existing disorders or influencing parameters that are prognostically significant in clinical settings. This justifies the need for more nuanced translational research.

## 11. ZEN Biosynthesis and Possibilities for Its Reduction

In research on zearalenone, it is becoming increasingly important not only to detect the effects of contamination, but also to understand and reduce the biosynthesis of the toxin by fungi. In this context, the work of Stakheev et al. is particularly interesting, as they demonstrated that compactin can almost completely inhibit ZEN production by the toxin-producing strain *Fusarium* culmorum without causing a comparable inhibition of fungal growth (Stakheev et al., 2022[38]).

The significance of this work lies in the fact that the authors did not limit themselves to describing the quantitative effect, but analysed changes in the expression of genes involved in ZEN biosynthesis and regulation. The observed strong reduction in the expression of genes such as PKS4, PKS13 and ZEB1 suggests that zearalenone production is much more susceptible to intervention at the regulatory level than previously assumed (Stakheev et al., 2022[38]).

## 12. Enzymatic Degradation of Zearalenone

This scientific progress enables the transition from a purely reactive safety model to the more predictive one required for modern food management. In this context, the impact of climate change on fungal growth is no longer just an environmental observation, but a critical variable that influences the effectiveness of these new preventive strategies. Integrated disease management must therefore account for temperature and humidity patterns as dynamic factors in toxin biosynthesis.

The second important area of intervention research is biological detoxification. A review by Fang et al. on zearalenone lactonases showed that lactone ring hydrolysis is one of the most promising ways to reduce the biological activity of ZEN (Fang et al., 2022[14]). The significance of this mechanism lies in the fact that it directly interferes with the structure responsible for oestrogen like activity, thus not only removing the compound from the matrix, but also potentially reducing its toxic properties.

Even more important are studies identifying specific enzymes with beneficial functional parameters. An example is the study by Fruhauf et al., which describes ZenA bacterial lactonases with non-canonical structural features capable of hydrolysing zearalenone (Fruhauf et al., 2024[15]). From a scientific point of view, the most interesting aspect here is the combination of structural biology with application potential. The relationship between enzyme structure and its effectiveness against ZEN is becoming better understood, which provides a basis for designing more efficient biocatalysts.

However, enzymatic detoxification is not without its limitations. The key questions remain the toxicological significance of degradation products, their stability and the behaviour of the process under real-world conditions, not just in the laboratory. Nevertheless, this direction should be considered one of the most promising for future applications in feed and food technology.

## 13. Analytical Progress and the Importance of Reference Materials

As exposure assessments become more complex, the importance of measurement quality increases. It is no longer sufficient to have a method that can detect the presence of ZEN. Methods capable of simultaneously determining multiple mycotoxins, metabolites and different biological matrices are needed, as well as appropriate metrological infrastructure to ensure the comparability of results. In this context, the development of certified reference materials for complex matrices such as vegetable oils is of great importance (Xu et al., 2025[44]).

The work of Xu et al. on the development of a reference material for aflatoxins and zearalenone in mixed corn and peanut oil shows that the challenge is not only the sensitivity of the equipment, but the entire infrastructure for reliable measurement (Xu et al., 2025[44]). In laboratory practice, the result of a determination is only valid if it can be related to a properly characterised material, the measurement uncertainty can be estimated, and the repeatability of the determinations in a given matrix can be demonstrated. This is particularly important in official controls, scientific research and disputes concerning product compliance with legal requirements.

Analytical progress also applies to biomonitoring. The developing LC-MS/MS methods for the determination of multiple mycotoxins in human and animal urine provide the basis for a more realistic estimation of exposure and for combining food toxicology with laboratory medicine (Chen et al., 2023[3]). Advanced metabolomics approaches based on high-resolution mass spectrometry are also increasingly applied in biomonitoring studies of mycotoxins. These analytical strategies enable the detection of previously unknown metabolites and conjugated derivatives, providing a more comprehensive understanding of metabolic pathways and exposure patterns in humans (Malachová et al., 2014[30]; Owolabi et al., 2024[34]). In this sense, analytics is not only the technical background for ZEN research, but one of the most important conditions for its credibility.

## 14. Practical Implications for Food, Feed and Public Health

The data collected in recent years has clear practical implications. In the food sector, it means that ZEN therefore be considered not only as a parameter for the compliance of a single batch of product, but as a risk indicator requiring a broader context. The origin of the raw material, weather conditions, storage history, co-occurrence of other toxins and the possibility of masked forms become important. Responsible food safety management therefore requires a more predictive model than a purely reactive one.

Preventing fungal infection during crop production remains one of the most effective strategies for reducing ZEN contamination in the food chain. Agricultural practices such as crop rotation, the use of resistant crop varieties and appropriate storage conditions play a crucial role in minimizing fungal growth and mycotoxin accumulation in cereals. Crop rotation with non-host plants, effective crop residue management and the cultivation of resistant cultivars are widely recognized agronomic approaches that can significantly reduce the incidence of *Fusarium* diseases and associated mycotoxin contamination (Alisaac and Mahlein, 2023[1]).

Recent research has highlighted the importance of integrated crop management strategies aimed at reducing *Fusarium* infection and subsequent mycotoxin production. These strategies include optimized irrigation practices, appropriate fungicide application and improved post-harvest storage conditions. Integrated disease management combining resistant varieties, crop rotation and targeted fungicide use is considered one of the most effective approaches to limiting *Fusarium* infections and the accumulation of toxins such as ZEN in cereal crops (Kos et al., 2023[25]). Climate change may further influence the effectiveness of these preventive strategies, as variations in temperature, humidity and precipitation patterns can modify fungal growth dynamics and mycotoxin production in agricultural systems (Casu et al., 2024[2]).

In the area of feed and animal production, it is particularly important that the effects of chronic exposure may manifest themselves not only in the reproductive system, but also in metabolic parameters, production efficiency, milk quality and the overall health of animals (EFSA, 2017[10]; Falkauskas et al., 2022[12]; Wu et al., 2022[43]). This reinforces the argument for combining feed monitoring with animal biomonitoring, at least in problem herds.

From a public health perspective, it seems most important that the current assessment of ZEN exposure cannot be limited to a simple statement of compliance or non-compliance of food with the legal limit. A broader perspective is needed, covering cumulative exposure, the sensitivity of specific groups and the quality of determination methods. In this sense, research on ZEN is an example of a more general change in food toxicology: a shift from a simple contamination model to a real biological burden model.

## 15. Biomonitoring and Human Exposure

Human biomonitoring has become an essential tool for providing direct evidence of internal exposure. Building upon the methodological principles discussed in Section 4, recent studies rely on the detection of toxins or their metabolites in biological matrices such as urine, blood, or breast milk to evaluate the actual intake and metabolic processing of mycotoxins in the human body. After ingestion of contaminated food, ZEN is rapidly metabolized and excreted primarily in urine. Consequently, urinary biomarkers are widely used indicators of human exposure. The most frequently detected biomarkers include the parent compound ZEN and its metabolites α-zearalenol (α-ZEL) and β-zearalenol (β-ZEL), as well as their glucuronide conjugates. The presence of these compounds in biological fluids confirms that exposure to ZEN occurs in many populations worldwide (Habschied et al., 2021[21]; Cramer et al., 2025[5]).

Recent biomonitoring studies conducted in different regions of the world from the HBM4EU program in Europe to studies in Nigeria and China confirm that exposure is widespread and varies significantly based on dietary habits and geographical location (Wallin et al., 2015[41]; Kolossa-Gehring et al., 2023[24]; Namorado et al., 2024[31]).

Higher exposure levels have often been reported in some African and Asian populations, where maize and other cereals constitute major dietary staples and storage conditions may favor fungal contamination. Biomonitoring studies conducted in Nigeria and Cameroon have detected ZEN metabolites in a substantial proportion of urine samples, indicating frequent exposure to *Fusarium* mycotoxins (Ezekiel et al., 2014[11]; Cramer et al., 2025[5]). Similar findings have been reported in Asian countries, including China, where urinary biomarker analyses have shown detectable ZEN metabolites in both adults and children, particularly in populations with high consumption of maize-based foods (Fan et al., 2021[13]).

Overall, these studies demonstrate that human exposure to ZEN occurs globally, although the levels detected are usually relatively low. Nevertheless, continuous dietary exposure through contaminated cereal products remains a relevant public health concern, particularly for populations with high cereal consumption.

## 16. Final Conclusions

An update of the state of knowledge following the 2021 article leads to several key conclusions. Firstly, zearalenone remains one of the most important *Fusarium* mycotoxins affecting global food and feed safety. Its frequent occurrence in cereals such as maize and wheat continues to represent a significant concern, particularly because these crops are highly susceptible to infection by *Fusarium* species. Environmental factors such as temperature and humidity strongly influence fungal growth and mycotoxin production, and ongoing climate change may further modify the patterns of ZEN contamination in agricultural systems (Ropejko and Twarużek, 2021[36]).

Secondly, the overall risk picture has become considerably more complex. While the oestrogen-like activity of ZEN remains the most characteristic and best documented mechanism of toxicity, increasing evidence shows that its biological effects extend beyond endocrine disruption. Contemporary studies indicate that ZEN may also induce oxidative stress, mitochondrial dysfunction, apoptosis and immune modulation, contributing to multi-organ toxic effects. At the same time, the growing importance of biomonitoring and the recognition of modified or “masked” forms of ZEN have significantly expanded the understanding of exposure pathways and toxicological relevance (EFSA, 2016[8]; Falkauskas et al., 2022[12]; Ruan et al., 2022[37]; Huo et al., 2025[23]).

Thirdly, modern research is gradually shifting the focus from simple detection of contamination toward a more integrated analysis of mechanisms, biomarkers of exposure and potential intervention strategies. Human biomonitoring studies detecting urinary metabolites such as α-zearalenol and β-zearalenol confirm that dietary exposure occurs in many populations worldwide, even when concentrations detected in food are relatively low. At the same time, advances in analytical chemistry, particularly multi mycotoxin methods based on LC-MS/MS and high-resolution mass spectrometry, have greatly improved the detection of ZEN and its modified forms in both food matrices and biological samples. Emerging technologies such as biosensors and rapid screening systems may further enhance food safety monitoring in the future (Chen et al., 2023[3]).

Fourthly, significant progress has also been made in strategies aimed at reducing ZEN contamination. These include attempts to limit toxin biosynthesis by *Fusarium* species as well as the development of biological and enzymatic detoxification methods capable of transforming ZEN into less toxic compounds. Microbial degradation and enzyme-based approaches are particularly promising, although adsorption-based feed additives and improved processing technologies remain important practical tools for reducing mycotoxin bioavailability in animal production (Fang et al., 2022[14]; Stakheev et al., 2022[38]; Fruhauf et al., 2024[15]).

Reliable exposure assessment requires not only sensitive analytical techniques but also appropriate reference materials, standardized protocols and well characterized measurement uncertainty to ensure comparability of results between laboratories and studies (Chen et al., 2023[3]; Xu et al., 2025[44]). Finally, all aforementioned advances lead to a necessary shift in perspective: ZEN should no longer be viewed as an isolated toxin present in a single product but rather as part of a dynamic system of environmental, metabolic, analytical and health related interactions. This integrated perspective, supported by the analytical and toxicological evidence presented in this review, allows for a realistic assessment of its significance and supports the development of effective strategies to reduce the risks associated with ZEN exposure in the food chain.

## Declaration

### Author contributions

Conceptualization, G.P., M.T.; Methodology, M.T.; Formal analysis, G.P., M.T.; Writing - original draft preparation, G.P.; Writing - review and editing, G.P.; Supervision, M.T.; Project administration, M.T. All authors have reviewed and agreed to the published version of the manuscript.

### Conflicts of interest

The authors declare no conflict of interest.

### Funding

Not applicable

### Data availability statement

Not applicable

### Informed consent statement

Not applicable

### Artificial Intelligence (AI) - assisted technology

The authors declare that no artificial intelligence (AI) technologies, including large language models (LLMs), chatbots, or AI-based content generation tools, were used to generate scientific content, analyze data, interpret results, or draft the manuscript. All scientific data, analyses, and conclusions were developed exclusively by the authors.

ChatGPT was used solely to improve the graphical presentation of the manuscript, while DeepL Translator was used exclusively for language refinement and proofreading. These tools did not contribute to the scientific content, interpretation of the results, or the original intellectual content of the manuscript. The authors take full responsibility for the accuracy, originality, and integrity of the submitted work.

## Figures and Tables

**Figure 1:**
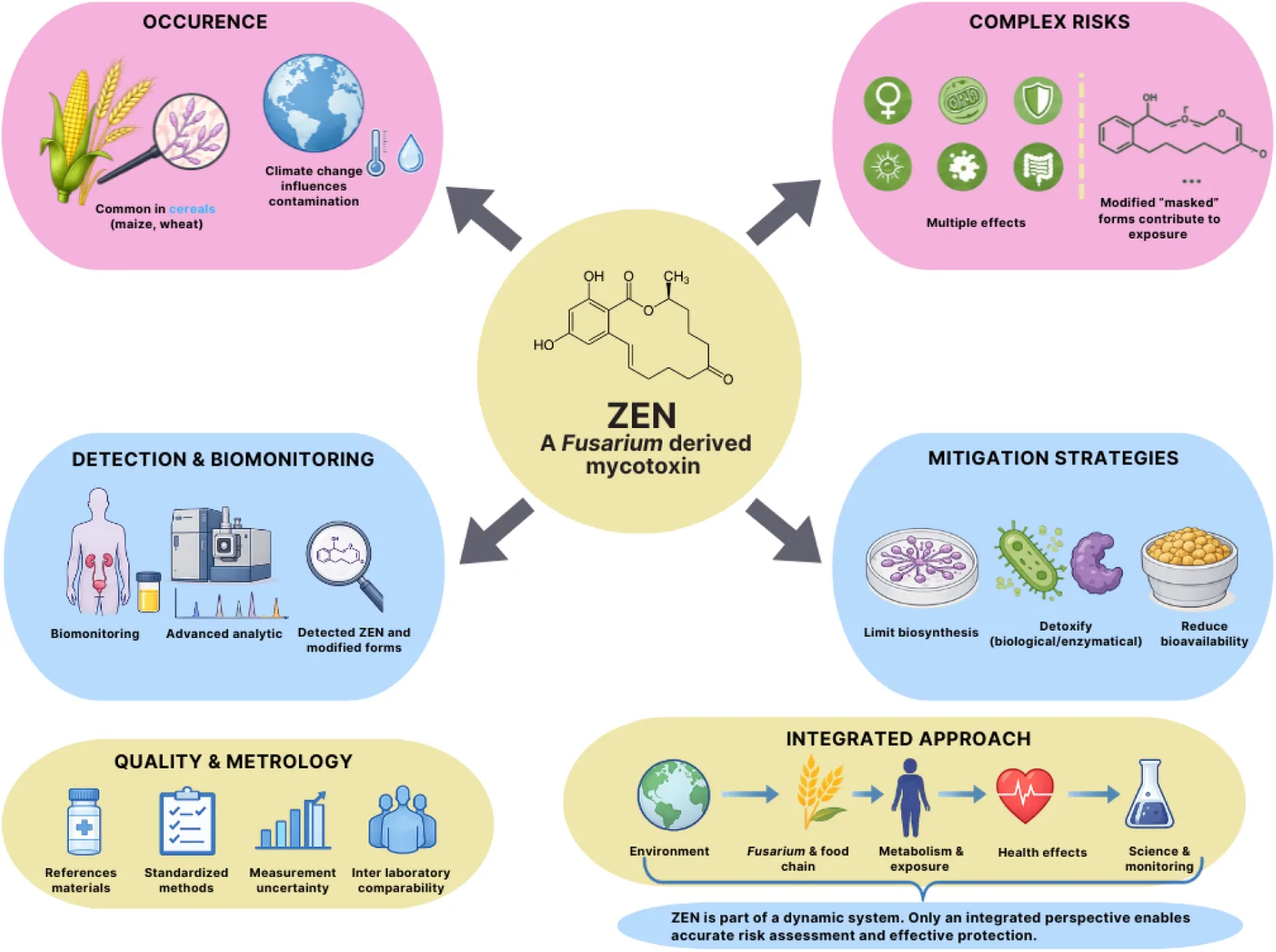
Graphical abstract
